# ROCK1 and LIMK2 Interact in Spread but Not Blebbing Cancer Cells

**DOI:** 10.1371/journal.pone.0003398

**Published:** 2008-10-14

**Authors:** Kerry F. Shea, Claire M. Wells, Andrew P. Garner, Gareth E. Jones

**Affiliations:** 1 Randall Division of Cell & Molecular Biophysics, King's College London, London, United Kingdom; 2 AstraZeneca, Cancer and Infection Research Area, Macclesfield, United Kingdom; 3 Division of Cancer Studies, Kings College London, London, United Kingdom; Max Planck Institute of Molecular Cell Biology and Genetics, Germany

## Abstract

Cancer cells migrating within a 3D microenvironment are able to adopt either a mesenchymal or amoeboid mode of migration. Amoeboid migration is characterised by membrane blebbing that is dependent on the Rho effectors, ROCK1/2. We identify LIMK2 as the preferred substrate for ROCK1 but find that LIMK2 did not induce membrane blebbing, suggesting that a LIMK2 pathway is not involved in amoeboid-mode migration. In support of this hypothesis, novel FRET data demonstrate a direct interaction between ROCK1 and LIMK2 in polarised but not blebbing cells. Our results point to a specific role for the ROCK1:LIMK2 pathway in mesenchymal-mode migration.

## Introduction

Breast cancer metastasis depends on cell migration, a complex process regulated spatially as well as temporally by the Rho family GTPases Rho, Rac and Cdc42 [1]. These GTPases elicit a response to extracellular signals on the actin cytoskeleton through a variety of effector proteins. In a 3D microenvironment cancer cells can adopt both mesenchymal and amoeboid like migratory phenotypes [2]. Amoeboid migration is characterised by membrane blebbing [3]–[5] a specialised form of cell protrusion that is reversible and can occur during cell migration or during the initiation of cytokinesis [6]. Membrane blebbing has been shown to be induced by Rho effector protein ROCK [7] and amoeboid-like movement is completely dependant on the interaction between Rho and ROCK [2], [5].

ROCK-1 and ROCK-2 are serine/threonine kinases which have a number of cellular substrates including Myosin Light Chain and LIM Kinase (LIMK) [8]. ROCK-dependent migration of cancer cells is known to be driven by actomyosin contractions [9], [10]. However it is not known whether ROCK dependent cancer cell amoeboid locomotion requires a ROCK: LIMK interaction.

Activated LIMK proteins phosphorylate and inactivate the F-actin severing protein, cofilin and this provides an alternative mechanism for Rho-ROCK signalling to mediate its effects on the F-actin cytoskeleton [11]. ROCK-LIMK signalling is thought to promote retraction of neurites through regulation of cofilin activity [12]. In addition, a role for ROCK and LIMK proteins in the human epidermis has been identified [13]. The inhibition of cofilin activity by ROCK-LIMK appears to be required for cell compaction where a decrease in LIMK activity leads to an increase in cofilin activity and a decrease in cell compaction [13].

An increase in ROCK levels has been detected in several human cancers [14]–[16] and levels of LIMK-1 increase in invasive and metastatic breast and prostate cell lines [17], [18]. Therefore we sought to better understand the contribution of a ROCK: LIMK interaction to cancer cell migration by imaging the spatial interaction between ROCK and LIMK in breast cancer cells exhibiting both mesenchymal and amoeboid (blebbing) morphologies.

## Materials and Methods

### Antibodies and Reagents

Anti-ROCK1 was purchased from Transduction Laboratories, Anti-LIMK2, anti-phospho-LIMK1/2 (Thr508/Thr505) from Cell Signalling Technology. HRP-conjugated secondary antibodies from DAKO and Alexa-phalloidin from Molecular Probes. Expression plasmids encoding GFP, CFP, YFP and mRFP1 tagged LIMK1, LIMK2 and ROCK1 were generated using Gateway™ Technology (Invitrogen) and all plasmids were sequenced. The ROCK inhibitor Y27632 was purchased from Calbiochem.

### Cell Culture

MDA-MB231 cells were grown in DMEM (Sigma) supplemented with 10% FBS (Helena Biosciences), L-glutamine and 100 U/ml penicillin-streptomycin. Cells were transiently transfected using Lipofectamine 2000 transfection reagent according to the manufacturers protocol (Invitrogen).

### Phosphorylation assay

Cells were lysed into NP40 lysis buffer (1% v/v NP40; 50 mM HEPES ph7.5; 0.5% w/v sodium deoxycholate; 150 mM NaCl; 1 mM EDTA). Samples were resolved by SDS-PAGE and immunoblotted. Autoradiographs were scanned and quantitated using Adobe software. Mean and s.e.m. values were calculated from the data of 3 independent experiments.

### Immunofluorescence and analysis

Cells seeded on glass coverslips were fixed with 4% paraformaldehyde:PBS and permeabilised with 0.2% Triton X-100:PBS as previously described [19]. Cells were then incubated with TRITC-conjugated phalloidin for 1 h at room temperature. Images of cells were obtained using a Zeiss LSM510 confocal laser-scanning microscope (Welwyn Garden City, UK) and were processed in Adobe Photoshop 7.0™. The Student paired t-test was used to compare differences between groups. Statistical significance was accepted for P≤0.05

### FRET: FLIM Microscopy

Cells were microinjected with the appropriate plasmids 24 hours prior to fixing. The cells were then fixed as above and incubated with fresh sodium borohydride (1 mg/ml in PBS) to quench background fluorescence as previously described. FLIM was performed on a multiphoton microscope as previously described [19]. FLIM analysis to calculate GFP lifetime and FRET efficiency was performed using TRI2 software [19]. The number of pixels for each FRET efficiency value were obtained from TRI2 and normalized by dividing it by the sum of the pixels for that image. This normalized pixel count was averaged over six cells per condition and then plotted against the FRET efficiency to generate FRET efficiency histograms.

## Results

### ROCK1 phosphorylates LIMK1 and LIMK2 in breast cancer cells

**Figure 1:**
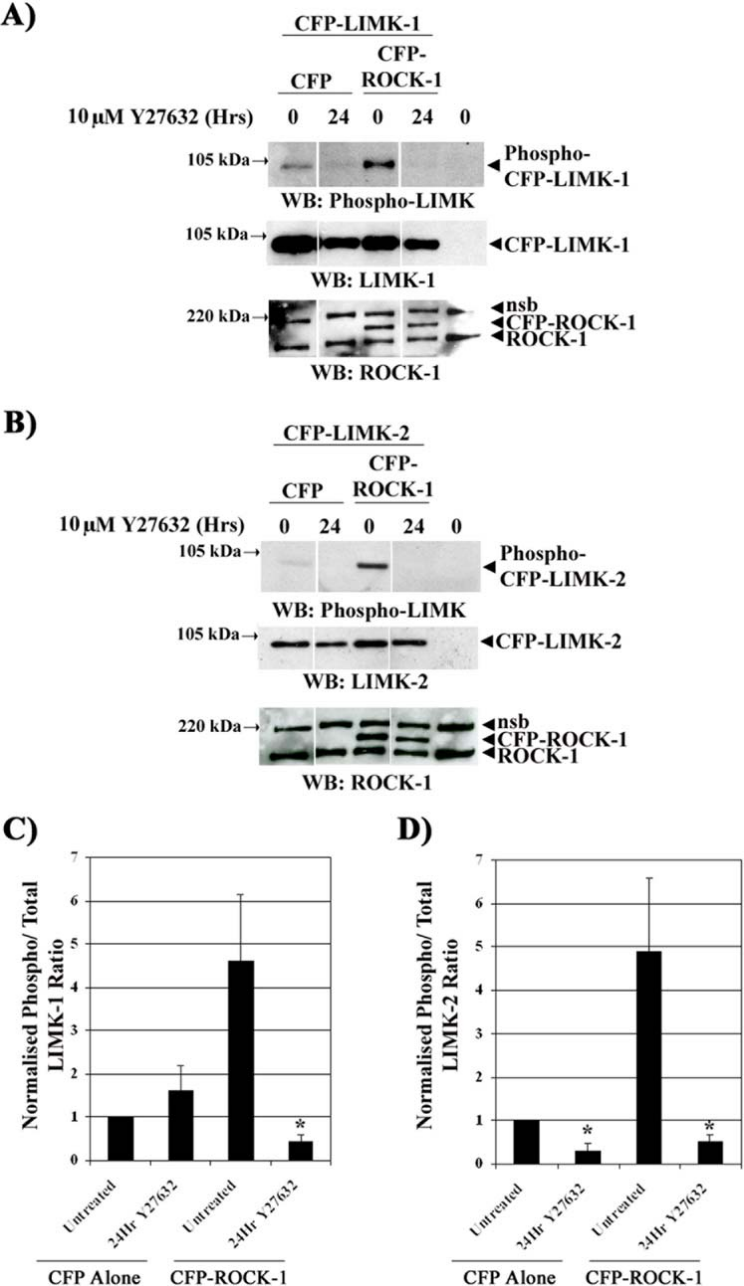
ROCK1 phosphorylates LIMK1 and LIMK2. A) and B) CFP-LIMK1 or 2 and either CFP-ROCK1 or CFP alone were transiently transfected into MDA-MB231 cells and treated with 10 µM Y27632 for 24 hours. The resultant lysates were immunoblotted using anti-phospho-LIMK, -LIMK1, -LIMK2 and -ROCK1 antibodies. (nsb = non-specific binding of the anti-ROCK1 antibody to a protein/s at 220 kDa). C) Ratio of phospho/total LIMK1 CFP-LIMK1 and D) Ratio of phospho/total LIMK2. (* = P<0.05).

A number of laboratories have suggested that ROCK can phosphorylate and activate LIMK1 and LIMK2 [20]–[26] and thus we sought to establish if this is the same for MDA-MB231 cells. Pre-incubation with the ROCK inhibitor Y27632 reduced the level of LIMK2 phosphorylation in cells with endogenous and overexpressed CFP-ROCK1. In contrast, Y27632 reduces the ratio of phospho to total LIMK1 in cells with overexpressed CFP-ROCK1 but not in those with endogenous levels of ROCK protein (Fig. 1). Treatment of cells with Y27632 induced a small decrease in LIMK1 and LIMK2 overexpression levels (Fig. 1A and B). However, whilst LIMK2 phosphorylation is always sensitive to ROCK activity LIMK1 phosphorylation is only sensitive to ROCK activity when CFP-ROCK1 is overexpressed. Thus, our results demonstrate that overexpression of ROCK1 alters the level of phosphorylation of both LIMK1 and 2 but suggest that LIMK2 is the preferred substrate of ROCK1 in these cells.

### ROCK1 but not LIMK2 induces blebbing in breast cancer cells

**Figure 2:**
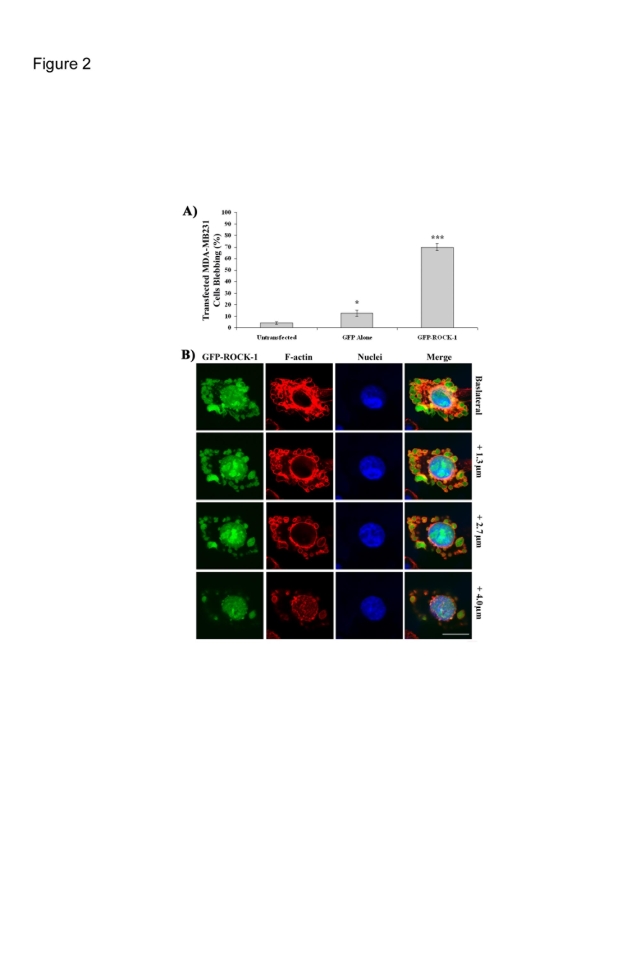
ROCK1 but not LIMK2 induces blebbing. A) MDA-MB231 cells were transfected with GFP-ROCK1 or GFP-LIMK2, fixed and stained with Alexa Fluor 594-Phalloidin and Dapi. 150 cells over 3 independent experiments were scored for visible blebbing+/−s.e.m. P<0.05; ***P<0.001. B) Representative images from varying optical slices of a blebbing cell overexpressing GFP-ROCK1. (Bar = 20 µm).

We found that whilst overexpression of GFP alone causes a small but statistically significant increase in the number of blebbing cells overexpression of GFP-ROCK1 induces a highly significant increase in the percentage of blebbing cells (Fig. 2A and B). Although not shown before in MDA-MB231 cells this has been reported in other cell types [9], [27]–[30]. In contrast overexpression of GFP-LIMK2 did not induce a high level of membrane blebbing, we also saw no indication of cell blebbing following overexpression of LIMK1 (data not shown). In all cases blebbing cells had an intact nucleus that did not fragment (Fig. 2A) indicating that this is not membrane blebbing associated with apoptosis [31].

### ROCK1 and LIMK2 do not interact in blebbing breast cancer cells

**Figure 3:**
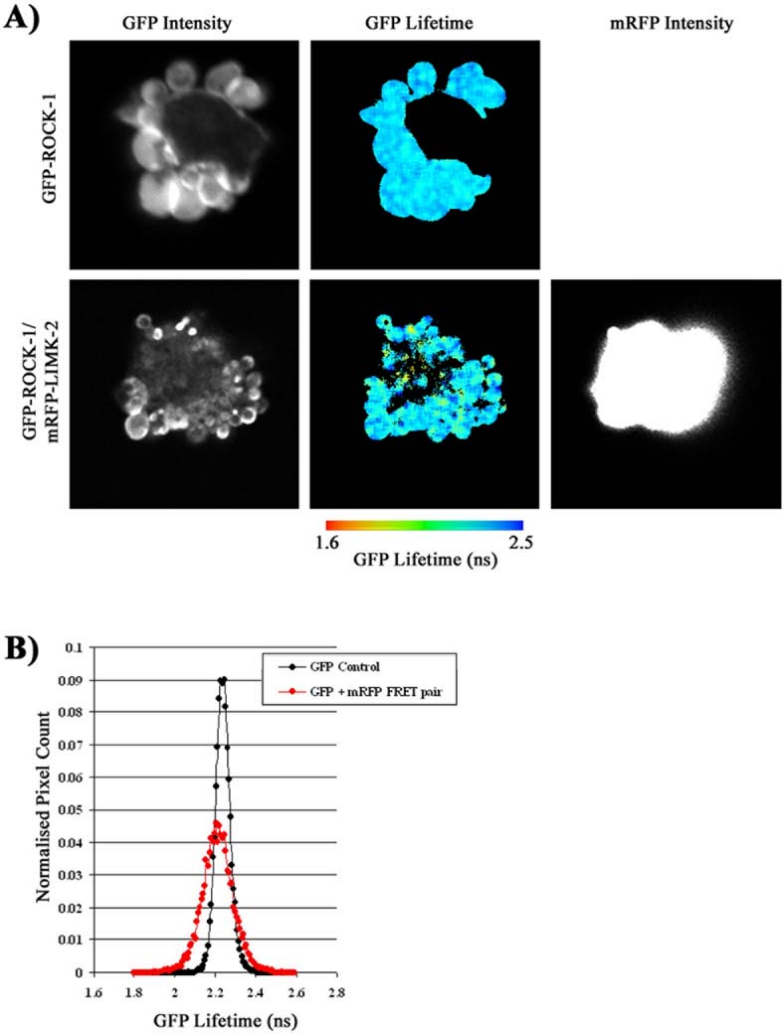
ROCK1 and LIMK2 do not interact in blebbing cells. MDA-MB231 cells were microinjected with GFP-ROCK1 and mRFP-LIMK2, fixed, imaged and analysed using FLIM microscopy and the TRI2 analysis programme. A) Images of the GFP lifetime and GFP and mRFP intensities across a typical blebbing cell was displayed for a cell expressing both the GFP-ROCK1 donor and the mRFP-LIMK2 acceptor and for comparison, only the GFP -ROCK1 donor. B) Histogram of the number of normalised pixel counts detected at each GFP lifetime. n = 9.

Our results show that there is an interaction between ROCK1 and LIMK2 but suggest that this interaction is not involved in membrane blebbing. We sought to confirm this hypothesis by directly imaging the interaction between ROCK1 and LIMK2 in blebbing and non-blebbing cells using FRET:FLIM microscopy. This method not only allows an interaction between ROCK1 and LIMK2 to be detected but also the localisation of such an interaction to be determined spatially across the whole cell. In order to compare the interaction of ROCK1 and LIMK2 in spread and blebbing cells we used microinjection to moderate the level of ROCK1 expression. Using this method the majority of cells, (63%), exhibited a spread/polarised morphology, with smaller number of blebbing cells (23%). In blebbing cells we detected no FRET between GFP-ROCK-1 and mRFP-LIMK-2 (Fig. 3).

### ROCK1 and LIMK2 interact in polarised breast cancer cells

**Figure 4:**
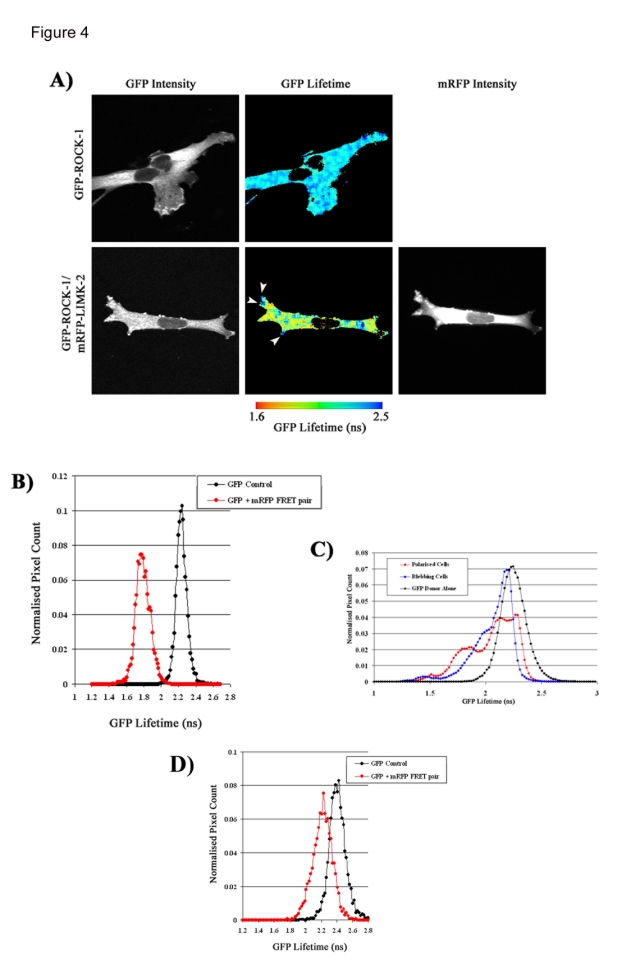
ROCK1 and LIMK2 interact in polarised cells. MDA-MB231 cells were microinjected with GFP-ROCK1 and mRFP-LIMK2, fixed, imaged and analysed using FLIM microscopy and the TRI2 analysis programme. A) Images of the GFP lifetime and GFP and mRFP intensities across a typical elongated cell was displayed for a cell expressing both the GFP-ROCK1 donor and the mRFP-LIMK2 acceptor and for comparison, only the GFP -ROCK1 donor. B) Histogram of the number of normalised pixel counts detected at each GFP lifetime. C) A histogram of the average number of normalised pixel counts detected at each GFP lifetime in cells expressing both GFP-ROCK1 donor and mRFP-LIMK2 acceptor in cells of elongated or blebbing morphologies was constructed along with cells expressing only the GFP-ROCK1 donor. 18 cells over three independent experiments were imaged for each time point. D) A Histogram of the number of normalised pixel counts detected at each GFP lifetime for cells expressing both GFP-ROCK-1 donor and mRFP-LIMK-2 acceptor in MDA-MB231 cells pre-treated with Y27632. n = 9.

Having established that ROCK1 and LIMK2 are not interacting in blebbing cells we analysed the localisation and interaction of ROCK1 and LIMK2 in spread cells. In spread cells the majority of LIMK2 and ROCK expression is localised in cytoplasm, but expression of both proteins can be detected in the nucleus (Fig. 4). In MDA-MB231 cells with a spread/polarised phenotype we detected a decreased GFP lifetime when ROCK1 and LIMK2 were co-expressed, showing that ROCK1 and LIMK2 interact in spread cells (Fig. 4). The GFP lifetime decrease is seen across the cell cytoplasm in a punctate distribution. In comparison, two polarised cells microinjected with only GFP-ROCK1 do not display any decrease in GFP lifetime (Fig. 4). There is no significant drop in GFP lifetime below control levels when cells expressing ROCK1 and LIMK2 are pre-incubated with the ROCK inhibitor Y27632 (Fig 4). Interestingly, in many cells there is a lack of any detectable interaction between ROCK1 and LIMK2 at the cell periphery (highlighted by arrowheads in Fig. 4).

## Discussion

Previous studies had not identified whether a ROCK: LIMK pathway contributed to the induction of membrane blebbing. We provide here for the first time evidence of a direct and specific interaction between ROCK1 and LIMK 2 in well-spread mesenchymal cells which is absent in rounded blebbing cells. Using FRET microscopy we found no interaction between ROCK-1 and LIMK-2 in cells that displayed a membrane blebbing phenotype, despite our own evidence that LIMK2 is the preferred ROCK substrate in these cells. Our results suggest that a ROCK1:LIMK2 interaction is not involved in the blebbing/rounded phenotype and would not be required for amoeboid migration. Indeed overexpression of LIMK2 does not induce membrane blebbing in cells. Recent reports suggest that cellular events downstream of ROCK activation are indeed separately coordinated though MLC and cofilin phosphorylation [32].

In contrast our FRET studies identified a direct interaction between ROCK1 and LIMK2 in concentrated foci in the cytoplasm of cancer cells with a mesenchymal morphology. The phosphorylation of LIMK-2 by ROCK-1 in the cell centre would increase the level of phosphorylated cofilin, thereby decreasing F-actin severing. This would stabilise the actomyosin filaments present in cell body and promote the generation of the contractile force necessary for tail retraction and cell migration [33]. Indeed, it has previously been shown that TGF-induced actin stress fibre formation is mediated by a ROCK-1/LIMK-2/cofilin pathway [26]. Recently, a ROCK: LIMK1 pathway was implicated in the co-ordination of cofilin activity at the plasma membrane of invasive rat mammary carcinoma cells [34], [35]. Our results point to a distinct function for LIMK1 and LIMK2 downstream of ROCK during breast cancer cell migration. We speculate that the interaction between ROCK1 and LIMK2 does not play a significant role in membrane blebbing associated cell migration nor in the regulation of cofilin phosphorylation at the cell periphery. Rather the interaction between ROCK1 and LIMK2 is restricted to the cell body of polarised well-spread cells where is contributes to the stabilisation of actomyosin filaments and the generation of contractile force through inactivation of cofilin.
